# Does the place of residence influence your risk of being hypertensive? A study-based on Nepal Demographic and Health Survey

**DOI:** 10.1038/s41440-023-01217-x

**Published:** 2023-03-08

**Authors:** Ishor Sharma, M. Karen Campbell, Yun-Hee Choi, Isaac Luginaah, Jason Mulimba Were, Juan-Camilo Vargas- Gonzalea, Saverio Stranges

**Affiliations:** 1 Department of Epidemiology and Biostatistics, Western University, London, ON Canada; 2 Department of Pediatrics, Western University, London, ON Canada; 3 Children’s Health Research Institute, London, ON Canada; 4 Lawson Health Research Institute, London, ON Canada; 5 Department of Obstetrics and Gynecology, Western University, London, ON Canada; 6 Department of Geography, Western University, London, ON Canada; 7 Department of Precision Health, Luxembourg Institute of Health, Strassen, Luxembourg; 8 The Africa Institute, Western University, London, ON Canada; 9 Department of family Medicine, Western University, London, ON Canada; 10 Department of Medicine, Schulich school of medicine and Dentistry, Western University, London, ON Canada

**Keywords:** Area level deprivation, Hypertension, Socio-economic status

## Abstract

Even though several studies have examined various risk factors for hypertension, residential influence is poorly explored especially in the low-income countries. We aim to investigate the association between residential characteristics and hypertension in resource limited and transitional settings like Nepal. A total of 14,652 individuals aged 15 and above were selected from 2016-Nepal Demographic and Health Survey. Individuals with blood pressure ≥140/90 mmHg or a history of hypertension (as identified by physicians/health professionals) or under antihypertensive medication were defined as hypertensive. Residential characteristics were represented by area level deprivation index, with a higher score representing higher level of deprivation. Association was explored using a two-level logistic regression. We also assessed if residential area modifies the association between individual socio-economic status and hypertension. Area deprivation had a significant inverse association with the risk of hypertension. Individuals from the least deprived areas had higher odds of hypertension compared to highly deprived areas 1.59 (95% CI 1.30, 1.89). Additionally, the association between literacy a proxy of socio-economic status and hypertension varied with a place of residence. Literate individuals from highly deprived areas were likely to have a higher odds of hypertension compared to those with no formal education. In contrast, literate from the least deprived areas had lower odds of hypertension. These results identify counterintuitive patterns of associations between residential characteristics and hypertension in Nepal, as compared with most of the epidemiological data from high-income countries. Differential stages of demographic and nutritional transitions between and within the countries might explain these associations.

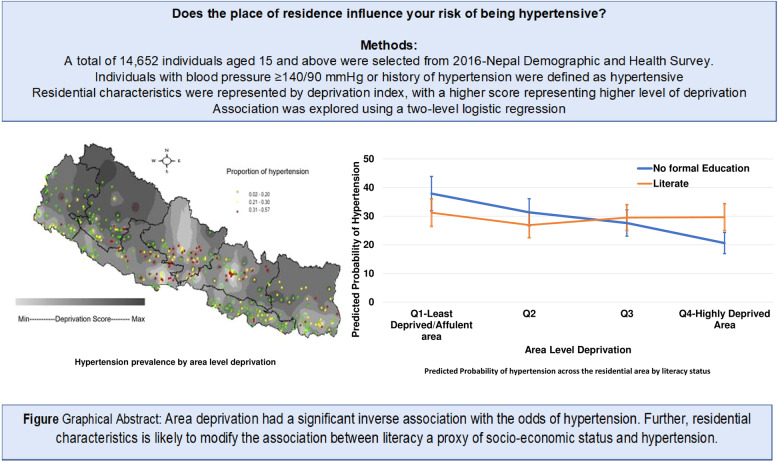

## Introduction

According to N. Krieger: “People literally embody, biologically, their lived experience, in societal and ecologic context, thereby creating population patterns of health and disease” [1]. This concept grounded in the social determinants of health identifies the disparity in health outcomes across the socioeconomically disadvantaged communities [2]. Therefore, understanding area level influence independently from individual factors is crucial for understanding population health [3–5].

Race/ethnic heterogeneity, unemployment rates, literacy levels, distribution of socio-economic status, environmental exposures, quality of the built environment, and geographical features (such as slope, and altitude) are often used to characterize these area-level influences on health [6]. However, these features are limited due to multicollinearity and may provide an incomplete picture of the underlying area level construct [7–9]. To alleviate these limitations partly, Townsend proposed a composite measure, the area level deprivation (AD), to characterize the residential characteristics, defined as “the relative disadvantage an individual or a social group experiences in terms of access and control over economic, material or social resources and opportunities” [10]. AD is often correlated with poor access to health services, food insecurity, health promoting-behaviors, and poorly built environments [11–14]. Individuals in socially and economically deprived areas are mostly at an elevated risk of adverse disease outcomes such as obesity, diabetes, and adverse mental health outcomes although it could vary across the regions [15–18]. Behavioral risk factors are relatively higher in such areas [19, 20]. However, most of these results are from the high-income western countries. Also, the association between individual factors and disease outcome is likely to vary across the residential areas [21, 22]. Residential characteristics may affect individuals’ decisions and options and, ultimately, their health differently. Health effects of living in the deprived areas could be different for individuals at different positions in the socio-economic ladder [23]. Despite increasing interest in the residential or area level influence on health, a very few studies especially in Low-and-Middle-Income-Countries (LMICs) have investigated on how residential characteristics are associated with disease outcome, and how individual and residential characteristics might interact to influence individual’s health.

Hypertension is an important preventable risk factor for morbidity and mortality [24]. Approximately one billion people suffer from hypertension globally and account for nearly 7.5 million annual deaths [25]. The estimated age-standardized global prevalence amongst adults (≥ 20 years) is approximately 31% [26]. Nepal has one of the highest prevalence of hypertension compared to its neighboring countries in South Asia [27]. Variations in the prevalence exists across the region ranging from 27.5% for the Gandaki Province to 13.9% for the Far Western Province. Across the ecological regions, the hilly region has the highest prevalence (22.6%) and the Himalayan lowest (16.2%) [28]. These spatial differences in hypertension prevalence call for a need to explore the underlying area level determinants. Although several prior studies have highlighted the heterogeneity in the prevalence of hypertension across the geographic regions of Nepal, none has explicitly examined the area level influence on such heterogeneity [29, 30]. Nonetheless, Nepal is undergoing a rapid epidemiological, demographic, and nutritional transition. These transitions are likely to vary across the geographic and administrative regions, which could have a significant influence on disease risk and distribution [21, 22]. Further, individual’s literacy status as a proxy measure for socio-economic status (literate representing higher socio-economic status) is seen to have a differential effect on disease risk across the residential area, which could be explained by individual decisions such as health seeking behaviors, and affordability [22, 31].

A comprehensive and in-depth exploration of the association between residential characteristics and hypertension could have important policy implications for identifying the target population, informing cost-effective strategies, and reducing health disparities across geographic regions. The study aims to explore the following:

1. How much of the variability in the prevalence of hypertension is attributed to place of residence? How much of this variability is explained by area level and individual level characteristics?
2. How is the place of residence is associated with the risk of hypertension?
3. Does the association between literacy status and hypertension vary with the place of residence?

Point of view

- Clinical relevance: Exploring the potential association between residential characteristics and hypertension would be vital in identifying the cost-effective health care interventions.
- Future direction: Understanding pathways on how the residential areas effect the chronic disease distribution in the rapidly transiting South Asian countries is warranted.
- Consideration for the Asian population: Counterintuitive patterns of associations between residential characteristics and hypertension is seen in most of the South Asian countries, as compared with the epidemiological data from high-income countries.

## Methods

### Data source

The study is based on the Nepal Demographic and Health Survey (NDHS)-2016, a nationwide cross-sectional survey that uses a multi-stage stratified cluster sampling [28]. The wards (lowest administrative unit) were selected as primary sampling units (PSUs). However, due to higher population density in urban areas, one enumeration area (EA) was selected from each ward. PSUs and EA were considered as cluster or the residential area. From each cluster approximately 30 households were selected. In total, 14,823 individuals with blood pressure measurement were available. Excluding pregnant mothers, 14,652 individuals were included for the final analysis.

### Individual level variables

Outcome variable: Hypertension was defined based on the guideline from the International Society of Hypertension [32]. An individual with systolic blood pressure ≥140 mmHg and/or diastolic blood pressure ≥90 mmHg was considered as hypertensive. Individual blood pressure was measured three times by UA-767F/FAC (A&D Medical) digital blood pressure monitor in the sitting position with the interval of 5 min. The average of the second and third measurements was taken into consideration and the first measurement was discarded. Additionally, participants with a medical diagnosis of hypertension or who indicated they were taking antihypertensive medication during the survey were considered as hypertensive regardless of their measured blood pressure.

### Area level characteristics

Construction and validation of the AD index were adopted from earlier studies [11, 33]. Briefly, a 15-item inventory was constructed based on community level literacy, employment status, household assets, household structures, geographical structure, and access to physical infrastructures at the cluster level. A one factor model obtained by exploratory factor analysis had standardized score (Mean = 100 and SD = 20) and ranged from 52 to a maximum of 146. The higher the index score, the greater the area level deprivation. Non-linearity in the association between AD and prevalence of hypertension was addressed by categorizing AD into quartiles. (Quartile 1) representing (least deprived areas), less deprived (quartile 2), moderate deprived (Quartile 3) and highest quartile (quartile 4) representing (highly deprived areas).

### Explanatory variables

Age (young adults (15 to <40 years), middle adults (40–60 years), elderly (60+ years)), and sex (Male/ Female) were considered as potential covariates. Lifestyle behaviors such as smoking/use of tobacco products and alcohol intake were classified as (Yes/No). Individuals were classified as literate (at least some level of formal education) and no formal education (without any formal education includes adult literacy). Body mass index (BMI) was classified using guidelines appropriate to South Asian population (underweight (<18.5 kg/m^2^), normal (18.5–22.9 kg/m^2^), Overweight/Obesity (>23 kg/m^2^) [34]. Household food insecurity (HFI) was assessed using the household food insecurity access scale (HFIAS) [35]. The HFIAS is a brief survey instrument to assess whether households have experienced problems with accessing food during the last 30 days. Households were categorized as food secure and food insecure based on the criteria by Food and Nutritional Technical Assistance project III, USAID [36]. Moderately food insecure and severely food insecure households were grouped together as food insecure households. Cronbach’s alpha reliability for HFIAs was found to be 0.82 indicating a high degree of internal consistency.

### Descriptive statistics

Mean and standard deviations were calculated for the continuous variable. Categorical variables were presented with percentages. Confidence intervals for prevalence (Normal distribution) were obtained by the bootstrapping with 1000 replications.

### Multilevel modeling

Due to the hierarchical nature of the data (individuals nested within clusters), a two-level logistic model was used to investigate the association between residential characteristics and hypertension. From each household, all the eligible individuals were selected for the blood pressure measure. On average there were around 2 individuals per household, so we considered individual variables as level-1 and AD as level-2 (residential area). Five models were developed. Model 1, the null model with no explanatory variables, includes only random intercepts and explores possible contextual effects, i.e., to what extent the observed variance in hypertension can be partitioned into individual and residential area. Model 2 includes the quartiles of AD and investigates the extent to which AD explain the area level differences. In Model 3 individual level non-modifiable variables such as (age, and sex) were added in the model 2. Model 4 included model 3 plus behavioral and social characteristics such as BMI, food insecurity, literacy status, alcohol intake, and smoking status. Interaction between residential characteristics and literacy status, sex were assessed (Model 5). The Wald statistic was used to test the significance of interaction terms. The results of the fixed effect parts of the models are presented in the form of ORs with 95% confidence intervals (CIs).

Area level variances were assessed from the random part of the model [37]. The variance partition coefficient (VPC) was used to operationalize the contextual phenomena and split overall variation into area/cluster level and individual level [38]. VPC is the proportion of total variance in the outcome attributable to the cluster or area administrative areas. The median odds ratio (MOR) describes the area level variance in the OR scale. It is defined as the median value of odds ratio between the area at the highest risk and the area at the lowest risk when randomly picking two areas [38]. Proportional Change in Variance (PCV) is the change in variance between the null model and the subsequent models [39]. Model fit indices were assessed using the loglikelihood for the nested model and Akaike Information Criteria (AIC) and Bayesian Information Criteria (BIC) for the independent models. Lower the AIC and BIC value better the fit of the model.

A sensitivity analysis was conducted to corroborate the study results based on the alternate definition of hypertension by American college of Cardiology/American Heart association (ACC/AHA) [40]. According to it, hypertension is defined based on systolic blood pressure ≥130 mmHg and/or diastolic blood pressure ≥80 mmHg. Additionally, participants with a medical diagnosis of hypertension or who indicated they were taking antihypertensive medication during the survey were considered as hypertensive regardless of their measured blood pressure. Conducting analysis using this definition, risk of hypertension at lower cut-offs could be explored.

Exploratory factor analysis and multilevel modeling were conducted using SAS software version 9.4 (SAS Institute, Cary, NC, USA). Spatial plotting was done with ArcGis-10.7 using the cluster level geographic positioning system co-ordinates obtained from the NDHS-2016 spatial data repository.

## Results

### Characteristics of the study population

In total 14,652 individuals within 383 clusters were available for the study. Cluster size ranged from minimum 10 to maximum 74 (average = 40) individuals. As seen in Table 1, the proportion who were elderly (age > 60 years) was higher (15.4%) for areas with moderate deprivation and least for the least deprived areas (11.9%). More than half of the study population was female across all the areas. Almost 80% were literate in the least deprived area while just 53% were literate in the highly deprived areas. Higher proportions of the individuals were smokers (12.3%) and were taking alcohol (2.9%) in highly deprived areas. Almost 35% were Overweight/Obese (>23 kg/m^2^) in the least deprived areas as compared to just 7.1% in the highly deprived areas. Almost 11% of the individuals were severely food insecure in the highly deprived areas while it was just 3.3% in the least deprived areas. Prevalence of hypertension was 27.5% for least deprived areas, however, it was only 17.9% in the highly deprived areas.

**Table 1:** Characteristics of the study population by Area Level Deprivation (NDHS-2016)

	Area level deprivation				
	Highly Deprived	Moderate Deprived	Less Deprived	Least Deprived	Total
Age (mean ± SD)	39.0 ± 18	40.1 ± 18	38.6 ± 18	36.7 ± 17	38.6 ± 17.6
15–39	1966 (55.8)	2004 (52.9)	2203 (57.2)	2182 (62.5)	8355 (57.0)
40–60	1015 (28.8)	1140 (30.1)	1069 (27.8)	895 (25.6)	4119 (28.1)
60+	541 (15.4)	644 (17.0)	579 (15.0)	414 (11.9)	2178 (14.9)
Sex					
Male	1490 (42.3)	1603 (42.3)	1613 (41.9)	1540 (44.1)	6246 (42.6)
Female	2032 (57.7)	2185 (57.7)	2238 (58.1)	1951 (55.9)	8406 (57.4)
Literacy					
No formal education	1659 (47.1)	1755 (46.3)	1461 (39.9)	698 (20.0)	5573 (38.1)
Literate	1863 (52.9)	2033 (53.7)	2390 (62.1)	2793 (80.0)	9076 (61.9)
Smoke (Last 1 year)					
No	3090 (87.7)	3330 (87.9)	3455 (89.7)	3246 (93.0)	13121 (89.6)
Yes	432 (12.3)	458 (12.1)	396 (10.3)	245 (7.0)	1531 (10.4)
Alcohol Intake					
No	3421 (97.1)	3729 (98.4)	3805 (98.8)	3472 (99.5)	14427 (98.5)
Yes	101 (2.9)	59 (1.6)	46 (1.2)	19 (0.5)	225 (1.5)
Body Mass Index					
Underweight (<18.5 kg/m^2^)	815 (23.1)	878 (23.2)	697 (18.1)	393 (11.3)	2783 (19)
Normal (18.5–22.9 kg/m^2^)	2457 (69.8)	2416 (63.8)	2483 (64.5)	1876 (53.7)	9232 (63)
Overweight/Obesity (>23 kg/m^2^)	250 (7.1)	494 (13.0)	671 (17.42)	1222 (35.0)	2637 (18)
Household Food Security (*n* = 14483)					
Secure	849 (24.2)	1610 (42.9)	1820 (48.2)	2437 (70.8)	6716 (46.4)
Mild insecure	309 (8.8)	528 (14.1)	529 (14.01)	304 (8.8)	1670 (11.5)
Moderately Insecure	1956 (55.8)	1404 (37.4)	1239 (32.8)	589 (17.1)	5188 (35.8)
Severely food insecure	393 (11.2)	212 (5.6)	189 (5.0)	115 (3.3)	909 (6.3)
Proportion of hypertension	631 (17.9)	874 (23.1)	885 (23.0)	961 (27.5)	3351 (22.9)
Total	3522 (24.1)	3788 (25.8)	3851 (26.3)	3491 (23.8)	14652

Figures in the parenthesis show the percentage

### Prevalence of hypertension

Overall prevalence of hypertension was 22.9%. Hypertension prevalence was higher amongst males (26.7 %) than females (20.1%). In total 11% (1641) of the individuals were aware of their hypertension status (history of hypertension or under medication), of which 51% had uncontrolled blood pressure during the time of the survey.

### Spatial distribution of area level deprivation and hypertension

**Fig. 1:**
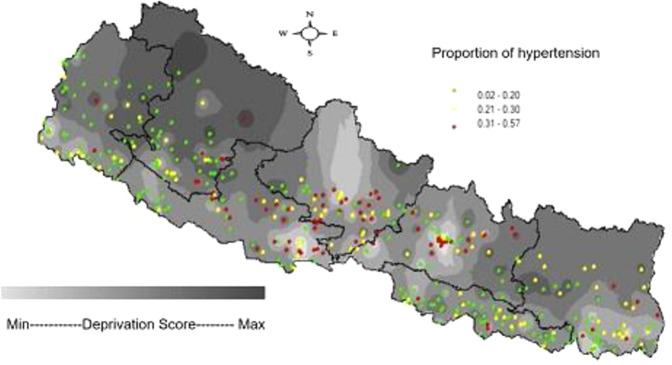
Area level deprivation and hypertension prevalence in Nepal- NDHS-2016

**Fig. 2:**
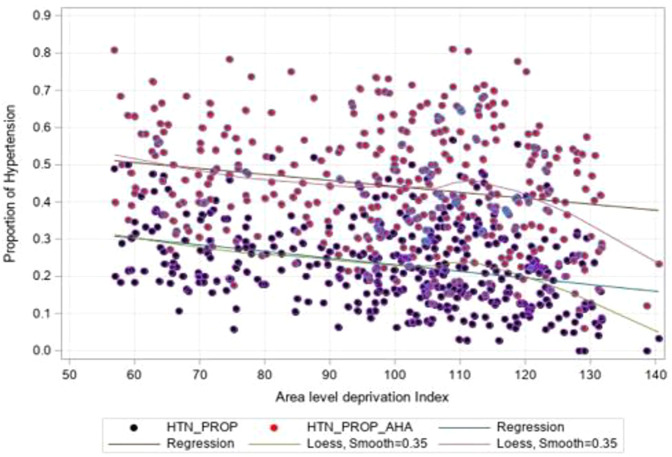
Area level deprivation index and the odds of hypertension

Figure 1 below shows the spatial distribution of an area deprivation and hypertension. The Northeast and North-Western region of the country have a higher level of deprivation. Higher prevalence of hypertension seemed to be more concentrated towards the lighter areas, meaning less deprived areas. A loess curve (Fig. 2) suggests that with an increasing deprivation index score, there is decreasing proportion of hypertension across the clusters, however, the relationship is not perfectly linear.

### Association between area level deprivation and hypertension

“As presented in Table 2, the variance explained by the area level characteristics is only 6.8% (Model 1). The remaining 93% of the variance could be due to individual differences. The MOR > 1.0 justified the need for multilevel analysis using the area level predictor [39].”

**Table 2:** Multilevel logistic regression analysis in Nepal (NDHS, 2016)

Variables	Model 1 (Null)	Model 2 OR (95% CI)	Model3 AOR (95% CI)	Model4 AOR (95% CI)	Model5
Fixed Effect					
Individual Factors					
Age (Ref: <40)			1	1	1
40–60			3.88 (3.52, 4.28)	3.21 (3.07, 3.80)	3.48 (3.12, 3.90)
60+			7.06 (6.29, 7.92)	7.98 (6.94, 9.18)	8.20 (7.12, 9.45)
Sex (Ref: Female)			1.34 (1.23, 1.46)	1.34 (1.22, 1.48)	1.34 (1.22, 1.49)
Smoke (Ref: No)				1.33 (1.16, 1.52)	1.33 (1.15, 1.52)
Alcohol (Ref: No)				1.13 (0.83, 1.55)	1.16 (0.85, 1.60)
Literacy (Ref: No formal education)				0.99 (0.89, 1.12)	1.02 (0.90, 1.15)
Food Insecurity (Ref: Food Secure)				0.97 (0.88, 1.07)	0.97 (0.88, 1.07)
BMI (Ref: Normal)					
Underweight				0.63 (0.55, 0.73)	0.64 (0.56, 0.73)
Overweight				2.61 (2.36, 2.88)	2.65 (2.40, 2,93)
Area level factor					
Area Deprivation					
Highly deprived		1	1	1	1
Moderate deprived		1.43 (1.19, 1.69)	1.41 (1.26, 1.85)	1.27 (1.05, 1.53)	1.29 (1.07, 1.56)
Less deprived		1.42 (1.19, 1.70)	1.52 (1.26, 1.85)	1.24 (1.02, 1.50)	1.21 (1.01, 1.47)
Least deprived		1.82 (1.53, 2.20)	2.25 (1.85, 2.73)	1.50 (1.23, 1.82)	1.59 (1.30, 1.89)
p-trend		0.001	0.001	0.002	0.002
Cross-Level Interaction					
Literate^*^High Deprived					1.62 (1.30, 2.02)
Literate^*^Mod. deprived					0.81 (0.99, 0.66)
Literate^*^Less deprived					1.10 (0.90, 1.34)
Literate^*^Least deprived					0.74 (0.59, 0.94)
Random effect					
Variance (SE)	0.24 (0.03)	0.21 (0.03)	0.26 (0.03)	0.22 (0.03)	0.18 (0.03)
VPC	6.8%	6%	7.3%	6.3%	5.2%
PCV	Ref	12.5%	-8.3%	8.3%	30.8%
MOR	1.59	1.55	1.60	1.56	1.50
Model fit Statistics					
Log Likelihood	15531	15497	13949	13235	13187
AIC	15535	15504	13967	13262	13221
BIC	15542	15515	14003	13318	13294

*SE* standard error, *VPC* Variance partition coefficient, *MOR* median odds ratio, *PCV* Proportional Change in Variance, *AIC* Akaike’s information criterion, *BIC* Bayesian information criteria

^*^P-value. Model 1: Null Model; Model 2: Unadjusted model; Model 3: Adjusted Model (Age, sex,); Model 4 Adjusted model (age, sex food insecurity, smoke, alcohol intake, literacy, and BMI categories); Model 5: including the interaction terms

In the unadjusted analysis, (Model 2, Table 2) there was an increase in the odds of hypertension with decrease in the area level deprivation index (*p*-trend = 0.001). Compared with the individuals in the highest quartile (highly deprived) individuals in the lowest quartile (least deprived) had higher odds of hypertension 1.82 (95% CI: 1.53, 2.20). Similarly, odds of hypertension in less deprived and moderately deprived areas were 1.42 (95% CI: 1.19, 1.70), and 1.43 (95% CI: 1.19, 1.69) respectively compared to highly deprived areas. Adjusting for age and sex (Model 3), the effect of deprivation levels was still significant. However, after adjusting for age, sex, food insecurity, alcohol intake, smoke, literacy, and BMI, the effect of AD on hypertension got attenuated but the association was still in the same direction (Model 4). Compared to the highly deprived quartile, the least deprived quartile had higher odds of hypertension 1.50 (95% CI 1.23, 1.82). Similarly, the odds of hypertension in less deprived and moderately deprived areas were 1.24 (1.02, 1.50), 1.50 (1.23, 1.82) respectively in compared to highly deprived areas.

### Interaction between individual factors and area level deprivation: Cross-Level Interaction

#### Sex and area level deprivation

There was no significant interaction between area level deprivation and sex; meaning that the association between sex and hypertension doesn’t vary by area level deprivation.

#### Effect of literacy levels on hypertension across the area with the level of deprivation

The association between literacy status and hypertension varied across the residential areas (*p*-interaction = 0.001) (Model 5, Table 2). Literate individuals compared to those with no formal education from the least deprived (i.e., more affluent) areas had lower odds of hypertension with OR = 0.74 (0.59; 0.94), while literate individual in highly deprived areas had higher odds of hypertension with OR = 1.62 (1.30, 2.02) in comparison to the individuals with no formal education.

**Fig. 3:**
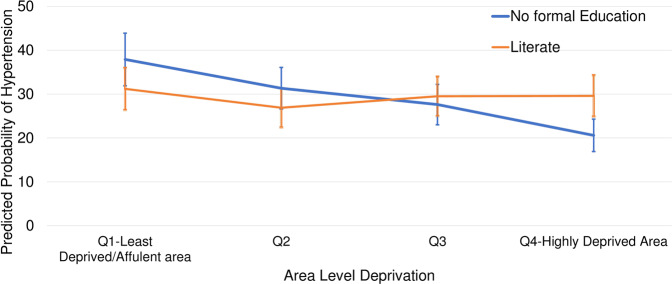
Predicted probability (%) of hypertension between the literacy groups across the place of residence. 2016 NDHS (with 95% Confidence Intervals)

To illustrate the interaction, we plotted the predicted probability of hypertension by literacy status across level of deprivation (Fig. 3). Individuals with no formal education from the highly deprived areas (4th Quartile) had the lowest predicted probability of hypertension of 20.6% (17%, 24.6%); however literate individuals from the same areas had predicted probability of 29.6% (25%, 35%). In contrast, the association was reverse in the least deprived areas where literate individuals had lower predicted probability of hypertension 31% (27%, 36%) compared to those with no formal education 38% (32%, 44%). Similarly, individuals with no formal education from the Q2 had slightly higher probability of hypertension 31.4% (26%, 36%) than the literate 27% (23%, 32%). For Q3 it was 29% for literate vs 28% with no formal education.

### Sensitivity analysis

According to the ACC/AHA criteria, the prevalence of hypertension was 44.1%. The higher prevalence was due to the lower cut-offs for systolic and diastolic blood pressure. The association between area level deprivation and hypertension was in line with AHA criteria (Fig. 2).

## Discussion

This is one of the first population-based studies to explore the potential influence of place of residence on hypertension in a low-resource and transitional settings such as Nepal. The findings showed that individuals from less deprived (more affluent) areas, may be at higher odds of hypertension compared to those from more deprived or resource poor settings. Residential characteristics are likely to modify the association between literacy status and hypertension.

Nepal has a significant topographic variation from 70 meters above sea level to the top of the Mount Everest (8848 meters). The difficult terrain of Nepal acted as a barrier for locomotive and communicative capability of people for centuries. Because of this, numerous ethnic groups (approximately 125 groups) with different lifestyles, dietary patterns, and languages were formed (Census Nepal, 2011). Although several studies have highlighted geographical and administrative variations of hypertension in Nepal, none of them really looked at these small area level variations and its interactions. Furthermore, the relatively high rates of hypertension in Nepal makes it important to understand and describe health inequalities, pinpoint the hotspots/clusters of health/disease outcomes that are essential to inform public health policy in the context of low-resource settings.

“With a relatively younger population, Nepal seemed to have a comparatively higher prevalence of hypertension compared to its neighboring countries [41, 42]. The socio-economic and the demographic transition with rapid reduction of poverty has significantly contributed to the increased prevalence of chronic conditions especially in LMICS [43–45]. According to NDTV India; 2013, poverty reduction in Nepal is significantly higher in Nepal than Bangladesh and India. This could partly explain the higher prevalence of hypertension in Nepal amongst its neighbors. In addition, per capita alcohol intake is relatively higher in Nepal than its counter parts Pakistan, Bangladesh, and Bhutan [46]. Similarly, tobacco use is relatively higher amongst the Nepalese population. Females in Nepal have the highest tobacco consumption rate in the entire South Asian region [47, 48]. Per capita salt intake in Nepal is 9.3 grams which is almost twice the recommended levels by the WHO [49].”

Only 7% of the variability in hypertension was attributable to residential area. Similar studies exploring area level influence on hypertension showed almost 14% of variability in Maharashtra, India [50], and 5% variability in Bangladesh [51]. A national study in India showed almost 28% of hypertension variability across districts [52]. Findings from these studies suggest a significant variation attributed to area level characteristics. However, they also vary with how the study site and area were defined. The fixed effects results are consistent with the other studies from LMICs. Compared to females, males seemed to have significantly higher risk, and this has been mostly attributed to lifestyle risk factors such as higher intake of alcohol, and smoking [53, 54]. In the current study, smoking was significantly associated with and increased odds of hypertension, although we see no significant association with alcohol intake.

Results of the current study are supported by several studies from LMICs suggesting individuals from the affluent communities at an increased risk of hypertension compared to those from resource poor settings [52, 55–57]. Based on the Popkin’s framework [58], like other LMICs, Nepal could be in the later stage of the nutritional and epidemiological transition characterized by increased accessibility and affordability of fats and refined food, increased intake of sugars and salts, sedentary behaviors, and higher prevalence of overweight and obesity [43, 55, 59–61]. These characteristics are particularly common in the affluent societies in LMICs [62, 63]. On the other hand, most studies from HICs and a limited number of the studies from LMICs show contrasting results [64, 65]. For instance, a US-wide cohort study showed the higher prevalence of hypertension amongst the deprived communities [66]. Similarly, studies from England showed adults from the most deprived neighborhoods had almost two times risk of obesity [67], and higher prevalence of chronic kidney diseases [68] in the deprived areas than in the least deprived areas. Residing in deprived areas in HICs is associated with elevated social stress [17], limited nutritive foods [69], higher access and intake of calorie rich processed and refined foods (60) [60], higher smoking rates, and sedentary lifestyle [70]. Additionally, deprived areas in HICs offer limited opportunity for recreational facilities and green spaces which is associated with increased risk of hypertension [13, 14, 71]. We need to be cautious with the result, as the outcome measure is also based on the self reported history of hypertension hence there is possibility of reporting bias. Individuals from the less deprived areas could have better health seeking behaviors [72] and likely to be identified with hypertension, leading to higher prevalence of hypertension in the affluent areas. Poor diagnosis of hypertension in the deprived areas might have led to underestimation of the true effect size.

In the current study, it is not surprising to see the differential effect of literacy status on hypertension across residential areas. We used literacy as a marker of an individual’s socio-economic status as it could represent one’s life experiences such as employment, and access to disposable income [73]. A nationally representative study across 76 LMICs showed that the countries with lower GDP per capita such as in Africa, and Southeast Asia there was a positive association between hypertension and education status; meaning hypertension was associated with higher literacy levels. In contrast, western pacific countries such as China, and Vietnam with relatively higher GDP showed inverse association between hypertension and education status [74]. The current study in Nepal found a non-significant positive association between educational status and hypertension. However, the association varied by the place of residence. In the highly deprived areas, literate individuals seem to have higher odds of hypertension when compared to those with no formal education. The association was reverse in affluent areas. This pattern could be explained by the “local social inequality model” which explains health disparity across socio-economic gradient within the residential area [23]. Individuals from the lower economic gradient in a residential area are more likely to be involved in low paying manual jobs. Furthermore, greater wealth in the area pushes up the prices of goods and services limiting its accessibility and affordability [23].”

In the context of Nepal, the traditional way of subsistence agriculture is the main source of livelihood for most individuals in deprived settings. In such settings, most individuals belonging to the lower economic gradient do not have formal education and any occupational activity besides traditional farming tend to be low paying manual jobs and construction work. Physical activity is a protective factor for hypertension [71]. Further, this group tend to have limited accessibility and affordability to refined and processed foods and are likely to have lower prevalence of overweight/obesity thus making them at lower risk of hypertension [75]. On the other hand, individuals from the higher socioeconomic ladder (literate groups) from the same resource poor settings are likely to have higher exposure and access to refined and processed foods and, sedentary lifestyles [76]. Thus, more susceptible to elevated risk of hypertension. However, this scenario is changing as we see an echoing of risk factors such as overweight/obesity from the wealthy to poor individuals even in resource poor settings [77, 78]. In contrast, literate individuals from the less deprived areas or affluent areas might be more concerned about their health, have better access to the healthy foods, making them at the lower risk of hypertension [23]. For instance, a recent study from urban population in India [62, 63], and Aleppo, Syria [79] suggested that the well-educated individuals from the affluent areas had lower prevalence of risk factors for chronic diseases.

Nepal has launched a multisectoral action plan with the goals of reducing preventable morbidity, avoidable disability and premature mortality due to NCDs [80]. Also, the government of Nepal, implemented the World Health Organization’s Package of Essential Noncommunicable Disease Interventions (WHO-PEN) [81]. This study portrays the different stages of epidemiological transition within Nepal which is undergoing rapid demographic and nutritional transition. Exploration of disease conditions at small area levels is important for geographically targeting public health resources for the prevention, diagnosis and effectively managing chronic conditions such as hypertension in resource poor settings. Consequently, this study which pinpoints geographic variations of hypertension at the smallest area level in Nepal is relevant for policy planning. For example, the high prevalence of hypertension seen in the urban areas and suburbs characterized by lower level of deprivation is noteworthy. Like most urbanizing countries, Nepal needs to pay particular attention to emerging health disparities in its urban context. Also, the study adds to our understanding of the within country variations in the epidemiological transition. For instance, being diagnosed with hypertension is likely to be higher amongst literates in the highly deprived areas, versus their counterparts least deprived areas. This signifies the underlying importance of deprivation as a potential explanation for the relatively high rates of hypertension in Nepal. The findings at the small area level could be fed in community-based hypertension program.

### Limitations

Results should be interpreted with caution recognizing limitations of the study. Definitive conclusions about cause and effect are limited as the study is based on cross-sectional data. Potential determinants like dietary patterns, family history, comorbidity status, income, and behavioral aspects were not available in the dataset, therefore affecting the ability to control for these in estimating the effect measure. Intake of alcohol and smoking was available only for a short period, which is likely to underestimate these potential risk factors’ true prevalence. The study population was based on a specific setting such as Nepal, hence findings may not be generalizable to other countries.

#### Perspective of Asia

Asian countries are going through a rapid socio-economic and nutritional transition. Burden of chronic diseases are on rise. Understanding the potential association between residential characteristics and hypertension would be an important aspect for designing the cost effective public health interventions.

## Conclusion

In conclusion, individuals from the most affluent areas of Nepal may be at an elevated risk of hypertension compared to those living in deprived areas. The association between literacy and hypertension is likely to vary across the residential area. Over-nutrition and changing lifestyle, driven by affluence and higher disposable income, could explain this counterintuitive association in the context of a country like Nepal which is undergoing a rapid socio-demographic and epidemiological transition. Based on the findings, there is a need for an area specific hypertension prevention program in Nepal to achieve its sustainable development goals on chronic disease prevention.

## Supplementary information

Supplementary Information
