# Supplementary material for: Does the place of residence influence your risk of being hypertensive? A study-based on Nepal Demographic and Health Survey
Source: Hypertens Res. 2023 Mar 8;46(6):1363–74. doi: 10.1038/s41440-023-01217-x (PMC10239727; doi:10.1038/s41440-023-01217-x)
Supplement: Supplementary file 1 — Supplementary Information [file 41440_2023_1217_MOESM1_ESM.docx]

## **Process of Area Level Deprivation Index Development**

Index development follows previous methodological works and approaches. [1-5] Briefly, the steps involved i) selection of relevant variables, ii) screening and assessment of variables, iii) variable reduction and extraction of the factors, and iv) assessment of validity and reliability.

### **Selection of the observed variables**

Variable selection was guided by the earlier studies, [6-10] availability of the variables in the dataset, and expert opinions. Based on these, a total of 26 aggregated and non-aggregated observed variables were selected that could explain the underlying construct; the Area-level deprivation. Aggregated variables were proportion or the average of the individuals/household’s characteristics at the area-level. These variables falls under six domains: ethic heterogeneity, education, employment, household assets, household structure, access to public and social infrastructures. Table 1 shows the detailed list of the variables.

Table 1: List of variables included for the construction of area level deprivation

|  | Variables for the area level deprivation |
| --- | --- |
| 1  2  3  4  5  6  7  8  9  10  11  12  13  14  15  16  17  18  19  20  21  22  23  24  25  26 | % of the disadvantageous/marginalized population  % of the dependent population  % of households with illiterate male/female  % of households with television  % of households with refrigerator  % of households with motorbike  % of households with cycle  % of households with car  % of households with radio  % of households with separate kitchen as cooking rooms  % of households with electricity  % of households with toilet  % of households with clean drinking water  % of households with shared toilet  % of households with rudimentary floor  % of households with rudimentary wall  % of households with phone  % of households with clean energy source  % of households with bank account  % of households with employed members  % of households exposed to mass media  % of household with soap  Average time to reach the nearest health facility (time in minutes)  Average time to reach the nearby motorable road (time in minutes)  Average time to reach to the nearby water source (time in minutes)  Altitude (in meters) |

### **Variables screening and assessment**

Variables with a low correlation (±0.30) in the correlation matrix and uniformly distributed across the clusters (>90%) were excluded. [11] **The Kaiser-Meyer-Olkin (KMO) measure of sampling adequacy and Bartlett's test of sphericity** were used to assess the suitability of data for index development. [12,13] The **KMO indicates the proportion of variance caused by the underlying factors and** ranges between 0 and 1, value above ≥0·7 is suggested. A significant Bartlett’s test suggests the correlation matrix of variables are significantly different from the identity matrix indicating the chosen variables are suitable for data reduction.

### **Variable reduction and factor extraction**

Principal component analysis (PCA) and Exploratory Factor Analysis (EFA) are commonly used techniques in construction of indices. As we are interested in the measurement of underlying latent construct; the area level deprivation, we selected EFA over PCA. [14,15]

EFA was conducted using iterated principal factor estimation due to lack of multivariate normality. [16] The number of factors extracted were based on eigenvalues > 1, visual inspection of the scree plot, and the conceptual meaning of the factor. Promax rotation was selected to assess factor loadings as the researcher expects some correlation between factors. Factor loading refers to the correlation of the variable with the latent structure. A more relaxed a priori criteria i.e., factor loadings <0.20 was used to exclude any variables from the factor. A minimum of 3 variables with high loading is considered for factor. Communality is the proportion of each variable variance that is explained by the underlying factor. High factor loadings and communality suggest a stronger relation between variables and the latent factor. The variables were then weighted by their factor loading.

### **Assessment of Index quality: validity and reliability**

Validity of the index was assessed by content, construct, and criterion validity. Content validity refers to the accurate representation of the underlying construct; the study especially sought to reflect the full scope of the area-level deprivation construct with the ADI. Confirmatory factor analysis (CFA) was conducted to test the construct validity of the index based on the factors obtained from EFA in the randomly sample (n=200). Maximum likelihood estimator with robust standard error was used. Root mean square error of approximation (RMSEA< 0.80), Comparative Fit Index (CFI ≥0.90) and the Tucker-Lewis Index (TLI ≥0.95) were used to assess model fit. [15] Modification indices was explored to identify model misfit areas. We also assessed for the place-based stability of the deprivation index (Invariance testing) by assessing factor loadings and its magnitude across three administrative regions (cluster, districts and ecological regions).[2] Criterion validity was assessed by correlating the newly constructed deprivation index with the 2018-MPI.[16] Reliability was assessed using Cronbach’s alpha.

Exploratory factor analysis was conducted using SAS version 9.4 (SAS Institute, Cary, NC, US), whereas confirmatory factor analysis was conducted in Mplus (Version 7, Muthén & Muthén, Los Angeles, CA, 2017). Spatial plotting was done using ArcGis-10.7 using the available cluster level geographic coordinate systems obtained from the Nepal DHS -2016 spatial data respiratory.

**Characteristics of the area level deprivation index**

A unifactorial model with 15-variables had the best fit to represent the underlying structure for area-level deprivation evidencing strong internal consistency (Cronbach’s alpha = 0.93). Standardized scores for index ranged from 58.0 to 140.0, with higher scores signifying greater area-level deprivation. Distribution of the area level deprivation including the health services utilization is provided in the figure 1.


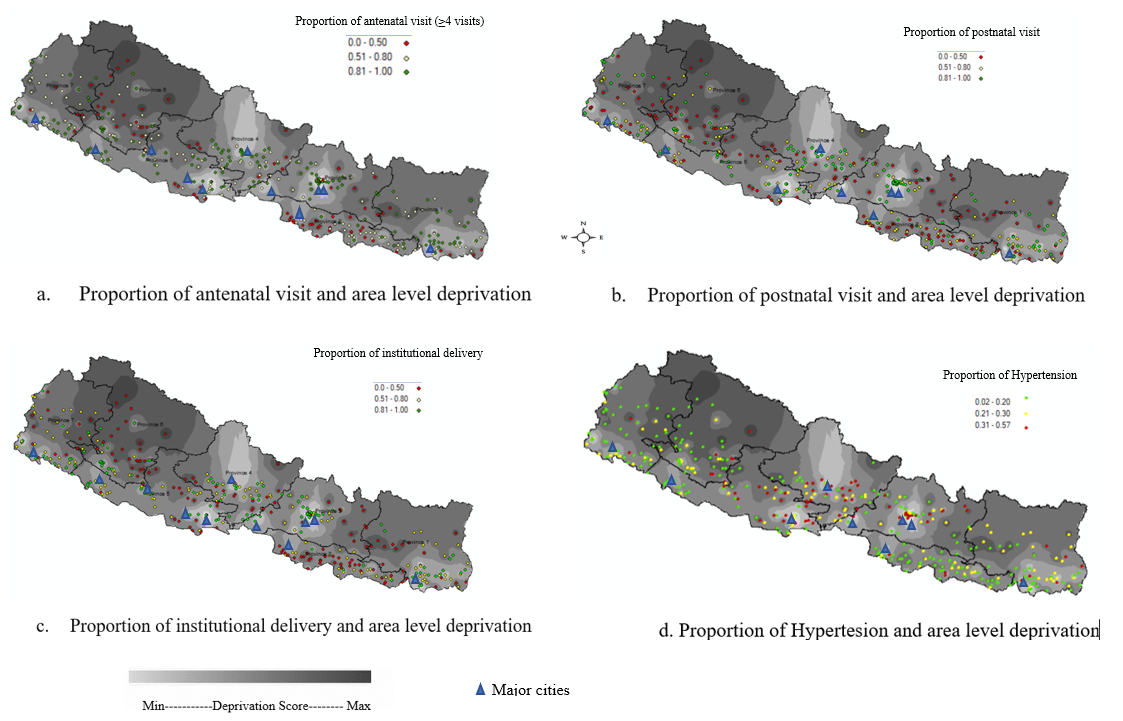


The North-Eastern and North-Western regions of the country appear to have a higher deprivation . The deprivation is in line with the urbanization, as urbanized areas appear to have a lower deprivation as compared to rural. We assessed if the correlation (Pearson’s Correlation (r)) between AD and health and health services utilization indicators are in line with the published studies. The proportion of Institutional delivery (r=-0.64), the proportion of hypertension (r= -0.32), and the proportion of obesity (r= -0.42) at the area level were negatively correlated with the deprivation score. Similarly, average time to reach nearby health facilities (r=0.30) and the proportion of ANC visit (<4 visits) (r=0.47) had a significant positive correlation with the deprivation score.

The newly constructed index showed relatively strong criterion validity with multi-dimensional poverty index (Pearson’s correlation coefficient=0.77) and relatively strong construct validity (Comparative Fit Index = 0.96; Tucker-Lewis Index= 0.94; standardized root mean square residual = 0.05; Root mean square error of approximation= 0.079). The factor structure was relatively consistent across different administrative regions.

Area level deprivation index was constructed and assessed for its validity and reliability. The index provides an opportunity to explore the area-level influence on disease outcome and health disparity.

**Table 1** Factor loading and shared variance (communality) for 15-variables at three different geographic levels (Cluster/area, districts, and sub-regional levels)

| Area level variables | Region | | | |
| --- | --- | --- | --- | --- |
|  | Cluster | | District | Sub-region |
|  | Factor loading | Communality | Factor loading | Factor loading |
| Proportion of households with illiterate population | 0.71 | 0.50 | 0.57 | 0.28 |
| Proportion of eligible population employed | 0.69 | 0.48 | 0.63 | 0.79 |
| Proportion of households with electricity | 0.46 | 0.21 | 0.46 | 0.71 |
| Proportion of households not exposed to mass media | 0.72 | 0.52 | 0.78 | 0.87 |
| Proportion of households without TV | 0.81 | 0.65 | 0.86 | 0.93 |
| Proportion of households without refrigerator | 0.85 | 0.72 | 0.88 | 0.95 |
| Proportion of households without motorcycle | 0.76 | 0.58 | 0.84 | 0.88 |
| Proportion of households with rudimentary floor | 0.91 | 0.82 | 0.93 | 0.94 |
| Proportion of households with rudimentary wall | 0.84 | 0.71 | 0.85 | 0.91 |
| Proportion of households without telephone | 0.61 | 0.37 | 0.64 | 0.61 |
| Proportion of households without clean energy source | 0.89 | 0.79 | 0.90 | 0.95 |
| Proportion of households without bank account | 0.63 | 0.40 | 0.72 | 0.91 |
| Proportion of households without soap for handwash | 0.71 | 0.50 | 0.73 | 0.75 |
| Average time required to reach nearby motorable road | 0.57 | 0.33 | 0.75 | 0.89 |
| Average time to reach to collect water | 0.48 | 0.23 | 0.45 | 0.63 |
| *Cronbach’s alpha reliability* | 93.0 |  | 0.94 | 0.97 |

**References**

1. Messer LC, Laraia BA, Kaufman JS, Eyster J, Holzman C, Culhane J, et al. The development of a standardized neighborhood deprivation index. J Urban Heal. 2006;83(6):1041-62.
2. Singh GK. Area Deprivation and Widening Inequalities in US Mortality, 1969-1998. Am J Public Health 2003;93(7):1137-43.
3. Watkins MW. Exploratory Factor Analysis: A Guide to Best Practice. J Black Psychol 2018;44(3):219–46.
4. Cole JC, Motivala SJ, Khanna D, et al. Validation of single-factor structure and scoring protocol for the Health Assessment Questionnaire-Disability Index. Arthritis Care Res 2005;53(4):536-42.
5. Williams B, Onsman A, Brown T. Exploratory factor analysis: A five-step guide for novices. J Emerg Prim Heal Care 2010;8(3).
6. Townsend P, Phillimore P, Beattie A.  Health and Deprivation: Inequality and the North. Routledge, London.1988.
7. Carstairs V, Morris R. Deprivation: Explaining differences in mortality between Scotland and England and Wales. Br Med J 1989;299(6704):886-89.
8. Statistics Canada. The Canadian Index of Multiple Deprivation - User Guide. Stat Canada Cat no 45-20-0001.2019.
9. Kirwa K, Eliot MN, Wang Y, et al. Residential proximity to major roadways and prevalent hypertension among postmenopausal women: Results from the women’s health initiative San Diego cohort. J Am Heart Assoc 2014;3(5):e000727.
10. Oka M, Yamamoto M, Mure K, Takeshita T, et al. Relationships between lifestyle, living environments, and incidence of hypertension in Japan (in men): Based on participant’s data from the nationwide medical check-up. PLoS One 2016;11(10):e0165313.
11. Beavers AS, Lounsbury JW, Richards JK, et al. Practical Considerations for Using Exploratory Factor Analysis in Educational Research. Pract Assessment, Res Eval 2013;18(6).
12. Watkins MW. Exploratory Factor Analysis: A Guide to Best Practice. J Black Psychol 2018;44(3):219–46.
13. Hoelzle JB, J. Meyer G. Exploratory Factor Analysis: Basics and Beyond. In: Handbook of Psychology, Second Edition 2012.
14. Costello AB, Osborne J. Best pr Best practices in explor actices in exploratory factor analysis: four or analysis: four recommendations for getting the most from your analysis. Pract Assessment, Res Eval 2005;10(7).
15. Hooper D, Coughlan J, Mullen MR. Structural equation modelling: Guidelines for determining model fit. Electron J Bus Res Methods 2008;6(1):53-60.
16. Oxford Poverty and Human Development Initiative (OPHI). Nepal’s Multidimensional Poverty Index. 2018. <https://mppn.org/nepal-multidimensional-poverty-index-2021>
